# Cardioprotective Effects of Salvianolic Acid A on Myocardial Ischemia-Reperfusion Injury *In Vivo* and *In Vitro*

**DOI:** 10.1155/2012/508938

**Published:** 2011-06-25

**Authors:** Huaying Fan, Liu Yang, Fenghua Fu, Hui Xu, Qinggang Meng, Haibo Zhu, Lirong Teng, Mingyan Yang, Leiming Zhang, Ziliang Zhang, Ke Liu

**Affiliations:** ^1^School of Pharmacy, Yantai University, Yantai 264005, China; ^2^College of Life Sciences, Jilin University, Changchun 130012, China; ^3^Laboratory of Pharmacology, Shandong Target Drug Research Co. Ltd., Yantai 264005, China; ^4^Ministry of Education Key Laboratory of Bioactive Substances and Resources Utilization of Chinese Herbal Medicine and Ministry of Health Key Laboratory of Biosynthesis of Natural Products, Institute of Materia Medica, Chinese Academy of Medical Sciences & Peking Union Medical College, Beijing 100050, China; ^5^School of Basic Medical Science, Beijing University of Chinese Medicine, Beijing 100029, China

## Abstract

Salvianolic acid A (SAA), one of the major active components of Danshen that is a traditional Chinese medicine, has been reported to possess protective effect in cardiac diseases and antioxidative activity. This study aims to investigate the cardioprotection of SAA *in vivo* and *in vitro* using the model of myocardial ischemia-reperfusion in rat and hydrogen peroxide (H_2_O_2_)-induced H9c2 rat cardiomyoblasts apoptosis. It was found that SAA significantly limited infarct size of ischemic myocardium when given immediately prior to reperfusion. SAA also significantly suppressed cellular injury and apoptotic cell death. Additionally, the results of western blot and phospho-specific antibody microarray analysis showed that SAA could up-regulate Bcl-2 expression and increase the phosphorylation of proteins such as Akt, p42/p44 extracellular signal-related kinases (Erk1/2), and their related effectors. The phosphorylation of those points was related to suppress apoptosis. In summary, SAA possesses marked protective effect on myocardial ischemia-reperfusion injury, which is related to its ability to reduce myocardial cell apoptosis and damage induced by oxidative stress. The protection is achieved via up-regulation of Bcl-2 expression and affecting protein phosphorylation. These findings indicate that SAA may be of value in cardioprotection during myocardial ischemia-reperfusion injury, which provide pharmacological evidence for clinical application.

## 1. Introduction

Ischemia-reperfusion (I/R) injury, a general healthy problem, is due to blood restoration after a critical period of coronary artery obstructions. It associates with a series of clinical problems such as thrombolysis, angioplasty, and coronary bypass surgery [1, 2]. Reperfusion damage is thought partly to be from oxidative stress, which could be partially prevented by antioxidants and free radical scavengers [2]. Apoptosis is a significant cellular mechanism responsible for ischemia-reperfusion injury in myocardium, and oxidative stress is a well-known factor promoting apoptosis [3, 4]. Therefore, reduction of apoptosis caused by oxidative stress could be an effective therapy for attenuation of ischemia-reperfusion injury. 

**Figure 1:**
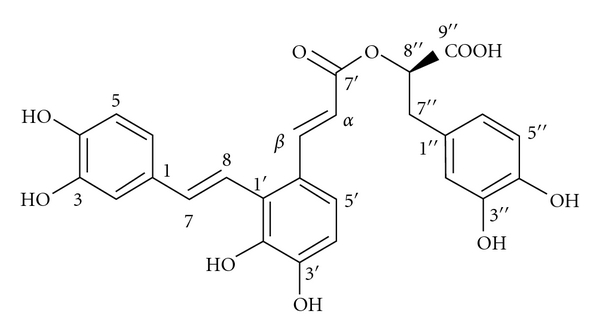
Chemical structure of salvianolic acid A (SAA). Molecular formula: C_26_H_22_O_10_; Molecular weight: 495.45.

The dried root of *Salvia miltiorrhiza *(Dan-Shen in Chinese) is one of the most popular traditional herbal medicines in some Asian countries and has been used extensively for the treatment of coronary artery diseases, angina pectoris, myocardial infarction, cerebrovascular diseases, various types of hepatitis, chronic renal failure, and dysmenorrhea and also to improve microcirculation in human body [5]. Research interest directed toward the water-soluble components of *S. miltiorrhiza *in recent years was initially motivated by its usage as a water decoction in some Chinese prescriptions and as injection for the treatment of cerebral and coronary vascular diseases [6]. Caffeic acid derivatives occur as the major water-soluble components of *Salvia miltiorrhiza*. To date, altogether twenty-five caffeic acid derivatives have been isolated from* S. miltiorrhiza *and have their structures elucidated through chemical and spectroscopic methods. This class of compounds includes caffeic acid monomers and oligomers, and the latter are also called depsides or salvianolic acids in the literature [7]. Salvianolic acid A (SAA, the chemical structure is presented in Figure 1) can be structurally divided into three caffeic acid units and has shown the most potent protective action against peroxidative damage to biomembranes among the different salvianolic acids [8]. In recent years, there is growing interest in SAA due to its potent bioactivities. Investigators have found that SAA possesses protection against ischemia-induced injury in a variety of experimental models and treatment with SAA was effective in models of acute myocardial ischemia in rats and ischemia-reperfusion-induced injury in isolated rat heart [9–11]. Although considerable evidence demonstrates the cardiac protective effect of SAA, its effect on ischemia-reperfusion-induced injury *in vivo* and the cardiac protection against cell apoptosis induced by oxidative stress remain largely unknown. 

According to our pharmacokinetic studies (unpublished data), SAA attained higher level in myocardial tissue than in other organs 1 h after intravenously administration. Given its potent pharmacological action and the common role of apoptosis and myocardial ischemia-reperfusion injury, SAA might be a promising cardioprotective agent. In the present, H_2_O_2_, the direct free radical donor in oxidative stress injury, is used to mimic oxidative stress in ischemia-reperfusion injury *in vitro*. Thus, the aim of this study was to assess SAA's cardioprotection by using experimental models of ischemia-reperfusion injury *in vivo* and hydrogen peroxide- (H_2_O_2_-) induced H9c2 rat cardiomyoblasts injury.

## 2. Methods

### 2.1. Animals

Male Sprague-Dawley rats (weight approximately 300 g) were purchased from Beijing Vital River Laboratory Animal Technology Co. Ltd., China (certificate no. SCXL (Jing) 2007-0001). Animals were acclimated for at least 1 week at a temperature of 24 ± 1°C and humidity 55 ± 5%. The animals were maintained with free access to standard diet and tap water. The experimental procedure was approved by the local Committee on Animal Care and Use.

### 2.2. Regents and Materials

 2,3,5-triphenyltetrazolium chloride (TTC) and Evans blue were products of Sigma Chemical Co. (USA). Dulbecco's Modified Eagle's Medium (DMEM) was purchased from Invitrogen Corporation (USA); fetal bovine serum (FBS) was purchased from Sijiqing Biological Engineering Materials Co. Ltd. (Hangzhou, China); trypsin and 3-(4,5-dimethylthiazol-2-yl)-2,5-diphenyltetrazolium (MTT) were products of Amresco Corporation (USA). Anti-Bax and anti-Bcl-2 were obtained from Cell Signaling Technology (USA). Penicillin, streptomycin, and antiactin were purchased from Beyotime Institute of Biotechnology (Jiangsu, China). Phosphospecific protein microarray (YT-PAP247) was a product of Full Moon BioSystems (USA). All chemicals used were of analytical grade.

### 2.3. *In Vivo* Myocardial Ischemia-Reperfusion Protocol and Evaluation of Infarct Size

Rats were anesthetized with pentobarbital sodium (45 mg/kg, i.p.). Under artificial ventilation with a rodent ventilator, a left thoracotomy was performed. Left anterior descending coronary artery (LAD) was surgically occluded for 90 min through ligation with a suture followed by coronary reperfusion through release of the tie. Coronary occlusion was confirmed through elevation of the ST segment on the ECG. The LAD was reperfused by untying the thread. The chest was then closed and the rats were monitored in the animal facility for 24 h. Sham-operated rats were subjected to identical treatment without tying the coronary ligature. Rats in control group did not have the operation. 

At the end of 24 h reperfusion, all rats were anesthetized with pentobarbital sodium. The left coronary artery was occluded by the silk suture in the same location as before. The abdomen was opened, and 5 mL of Evans blue dye (1% in saline) was injected into the vena cava to delineate the ischemic zone from the nonischemic zone. The heart was rapidly excised and cross-sectioned into 6 sections, which were then incubated in 1.0% 2,3,5-triphenyltetrazolium chloride for 10 min at 37°C to demarcate the viable and nonviable myocardium within the risk zone. The infarct size was determined as the ratio between the pixel of necrotic area and ischemic area at risk using Adobe Photoshop 7.0. 

SAA (5–40 mg/kg) dissolved in 10% saline was administrated intravenously within 10 min using syringe pump, beginning at 70 min of arterial occlusion and again at 1 h of reperfusion. The same amount of 10% saline was administered in the same manner to rats of the sham, vehicle, and control groups. 

### 2.4. Cell Culture and Treatment

The rat cardiomyoblasts line H9c2 was obtained from the Cell Bank of the Chinese Academy of Science (Shanghai, China). Cells were maintained in DMEM with 20% FBS, 100 U/mL penicillin, and 100 *μ*g/mL streptomycin at 37°C in a humidified atmosphere of 5% CO_2_. Experiments were carried out after cells were cultured for 24 h. Cells were pretreated with or without SAA for another 24 h, followed by incubation with H_2_O_2_ (120 *μ*M) for 1 h.

### 2.5. Cell Viability Assay and Measurement of SOD Activity

 Cells were plated at a density of 1.9 × 10^4^ cells/cm^2^ per well of 96-well plates. Cell viability was measured by MTT assay, through MTT labeling at a final concentration of 0.5 mg/mL for 4 h at 37°C. Viability was then evaluated by measuring the absorbance at 570 nm and 630 nm using Microplate Reader (BIO-TEK Instruments Inc.). For superoxide dismutase (SOD) activity assay, experiment was carried out with the corresponding commercial kit (Jiancheng; Nanjing, China) according to the manufacturer's instructions. Cells were plated at a density of 1.3 × 10^4^ cells/cm^2^ per well of six-well plates and pretreated with SAA for 24 h before H_2_O_2_ stimulation. After that, 500 *μ*L of DMEM per well were added into the plates and cells were cultured for 2 h. Then cells were washed once with phosphate-buffered saline (PBS). Five hundred microliters of PBS per well were added into the plates at −80°C in three repeated freezing and thawing cycles. Supernatant was collected by centrifugation at 5500 ×g for 5 min at 4°C for determination of SOD. 

### 2.6. Detection of Apoptosis with Annexin-PI

Cells were cultured at a density of 2.6 × 10^4^ cells/cm^2^ per plate. Cells were harvested with trypsin (0.25%) and centrifugation, washed twice with cold PBS and resuspended in 500 *μ*L binding buffer. Then the cells were incubated with 5 *μ*L Annexin V-EGFP (Jingmei Bio-Engineering Co., Ltd.; Shanghai, China) and 5 *μ*L propidium iodide (PI) for 15 min in the dark at room temperature. Fluorescence was measured on a flow cytometer at 488 nm and 530 nm.

### 2.7. Western Blot Analysis

 Cells were cultured at a density of 2.6 × 10^4^ cells/cm^2^ per plate. After treatment with H_2_O_2_ for 1 h, cells were washed once with cold PBS and then lysed with RIPA buffer (50 mM Tris, 150 mM NaCl, 1% Triton X-100, 1% sodium deoxycholate, 0.1% SDS, sodium orthovanadate, sodium fluoride, EDTA, and leupeptin) and 1 mM phenylmethylsulfonyl fluoride (PMSF) for 15 min on ice. Soluble proteins were collected by centrifugation at 5500 ×g for 5 min. The protein concentration in each sample was determined using a BCA protein assay kit (with BSA as a standard). For immunoblotting, 80 *μ*g of protein were loaded onto a 15% SDS-polyacrylamide gel electrophoresis (PAGE) and subsequently transferred onto a polyvinylidene difluoride (PVDF) membrane. Adequate transfer of protein was confirmed by Coomassie Blue staining of the gel and Ponceau Red staining of the membranes. Equal protein loading was confirmed by probing for *β*-actin. After blocking with 7% skim milk, the membranes were probed with respective primary antibodies (1 : 1000 dilution). The membranes were subsequently probed with horseradish peroxidase-conjugated antirabbit antibody (1 : 1500). Proteins were detected using enhanced chemiluminescence ECL Western blotting detection reagent, and bands were visualized by exposure to photographic film. Densitometric analysis of protein band was performed by using BandScan 4.0 software.

### 2.8. Phosphospecific Protein Microarray Analysis

Phosphospecific protein microarray was obtained from Full Moon Biosystems Inc. Protein microarray analysis was carried out using the protocol provided. The procedure used to process the array was as follows: 50 *μ*g of cell lysates in 60 *μ*L of reaction solution were labeled with 3 *μ*L biotin in 10 mg/mL N,N-dimethylformamide. 50 *μ*g of biotin-labeled proteins were diluted in 6 mL of coupling solution and labeled as “Protein Coupling Mix” before being applied to the array for conjugation. To prepare the antibody microarray, it was blocked with blocking solution for 45 min at room temperature, rinsed with Milli-Q grade water. Then the array was incubated with Protein Coupling Mix on an orbital shaker rotating at 35 rpm for 2 h at room temperature. Afterwards, the array slide was washed twice with 30 mL of wash solution for 10 min each, rinsed extensively with Milli-Q water, and then incubated with a Cy3-steptavidin (0.5 mg/mL) for 45 min in the dark at room temperature. This was followed by rinsing steps with Milli-Q water. After drying by centrifugation, the slide was scanned on a GenePix 4000B scanner (Axon Instruments, USA) and the images were analyzed with GenePix Pro 6.0. Fluorescence intensity of each array spot was quantified, and the mean value was calculated. For each treatment group, a phosphorylation signal ratio induction or reduction was calculated based on the following equation:

(1) $${\text{Phosphorylation}}_{\text{ratio}}=\frac{\text{phosphoA/unphosphoA}}{\text{phosphoB/unphosphoB}},$$

where phosphoA or phosphoB and unphosphoA or unphosphoB were signals of the phosphorylated and nonphosphorylated proteins from the experimental samples, respectively.

### 2.9. Statistical Analysis

 All data *in vitro* represent the mean of samples from three separate experiments. Results were expressed as mean ± standard deviation (mean ± SD). The significance of differences was analyzed by one-way ANOVA followed by Tukey's test. A value of *P* < .05 was considered statistically significant.

## 3. Results

### 3.1. Effect of SAA on Myocardial Infarct Size

**Figure 2:**
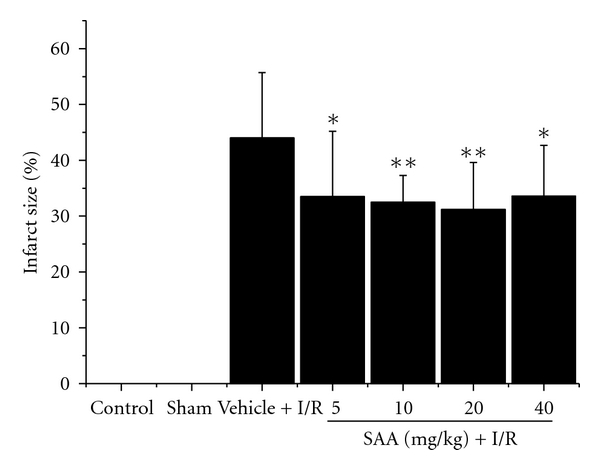
Effect of salvianolic acid A (SAA) on infarct size following cardiac ischemia-reperfusion. Rats were subjected to 90 min of ischemia and 24 h reperfusion. SAA (5, 10, 20, and 40 mg/kg) was intravenously administered 20 min before and 1 h after reperfusion using syringe pump, respectively. At the end of the reperfusion, the heart was excised and the ischemic zone was visualized using Evans blue dye. Necrotic tissue was evaluated by triphenyltetrazolium staining. Data are represented as mean ± SD of 12 animals for each group. *P < .05, **P < .01 versus vehicle. I/R: ischemia-reperfusion.

After 24 h of reperfusion, infarct size was 44.0 ± 11.7% of the area at risk (Figure 2). Intravenous administration of SAA at 5, 10, 20, and 40 mg/kg significantly reduced infarct size as compared with vehicle. The infarct size was 33.5 ± 5.7%, 32.5 ± 4.8%, 31.2 ± 8.4%, and 33.6 ± 9.1%, respectively.

### 3.2. Effect of SAA on H_2_O_2_-Induced Deficiency of Cell Viability

**Figure 3:**
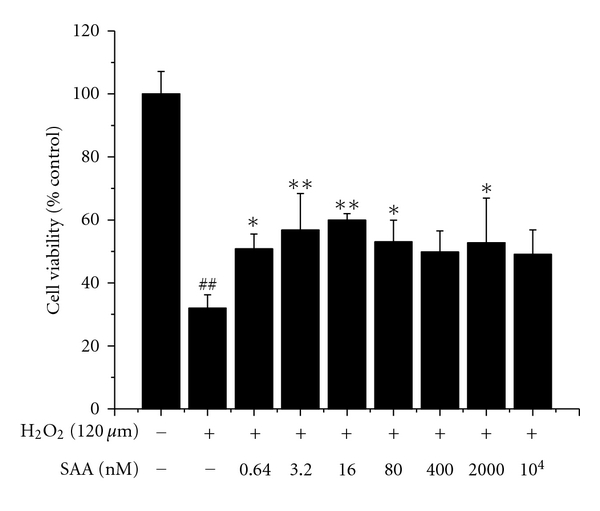
Salvianolic acid A (SAA) inhibits H_2_O_2_-induced deficiency of cell viability. H9c2 cells were pretreated with or without SAA (0.64–10^4^ nM) for 24 h prior to incubation of cells with H_2_O_2_ for 1 h. Data are represented as means ± SD of three independent experiments. ^##^ *P* < .01 versus control; *P < .05, **P < .01 versus H_2_O_2_.

 As shown in Figure 3, H9c2 cells viability fell to 32.0 ± 4.3% with exposure to 120 *μ*M H_2_O_2_ for 1 h. Pretreatment with different concentrations of SAA could attenuate H_2_O_2_ cytotoxicity. SAA at concentrations ranging from 0.64 to 16 nM significantly increased cell viability in a dose-dependent manner. The viabilities were 50.8 ± 4.7%, 56.7 ± 11.6%, and 59.9 ± 2.1%, respectively. The three concentrations were therefore used in subsequent *in vitro* studies.

### 3.3. Effect of SAA on SOD Activity in H_2_O_2_-Treated H9c2 Cells

**Figure 4:**
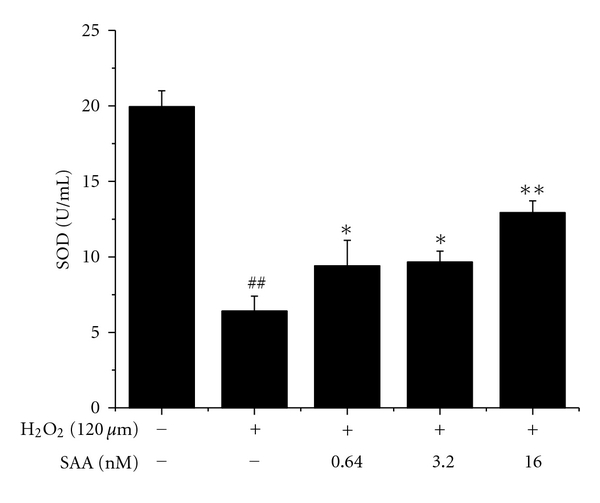
Effect of salvianolic acid A (SAA) on SOD activity in H9c2 cells treated with H_2_O_2_. H9c2 cells were pretreated with or without SAA for 24 h prior to incubation of cells with H_2_O_2_ for 1 h. SOD activity was measured according to the corresponding commercial kits. Data are represented as mean ± SD of three independent experiments. ^##^ *P* < .01 versus normal control; *P < .05, **P < .01 versus H_2_O_2_.

H_2_O_2_ treatment significantly decreased SOD activity. 0.64, 3.2, and 16 nM of SAA dependently increased SOD activity in cells, which showed protective effect against oxidative damage induced by H_2_O_2_ (Figure 4).

### 3.4. SAA Protects H9c2 Cells from H_2_O_2_-Induced Apoptosis

**Figure 5:**
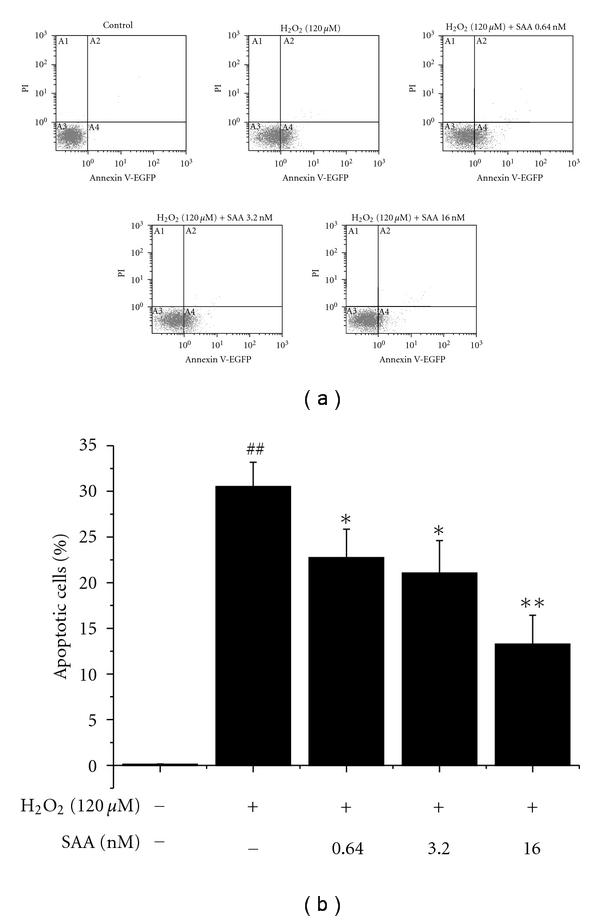
Salvianolic acid A (SAA) protects H9c2 cells from H_2_O_2_-induced apoptosis. FCM analysis with Annexin-V binding. H9c2 cells were pretreated with or without SAA for 24 h prior to incubation of cells with H_2_O_2_ for 1 h. Cells in region A4 represent early apoptotic cells. Data are expressed as mean ± SD of three independent experiments. ^##^ *P* < .01 versus control; *P < .05, **P < .01 versus H_2_O_2_. PI: propidium iodide.

 The antiapoptotic effect of SAA was confirmed by flow cytometric analysis: the phosphatidylserine translocation, which is considered to be a hallmark of early-stage apoptosis, assessed by Annexin V-EGFP binding. Flow cytometric analysis of Annexin V/PI staining of H9c2 cells showed that 30.5% of cells were in the early apoptotic stage when exposed to H_2_O_2_ for 1 h. After pretreatment with 0.64, 3.2, and 16 nM of SAA, 22.7%, 21.0%, and 13.3% of the cells were in the early apoptotic stage, respectively (Figure 5). It suggested that SAA could decrease H_2_O_2_-induced apoptosis in the early apoptotic stage to protect cardiomyoblasts.

### 3.5. Effect of SAA on Expression of Bcl-2 and Bax

**Figure 6:**
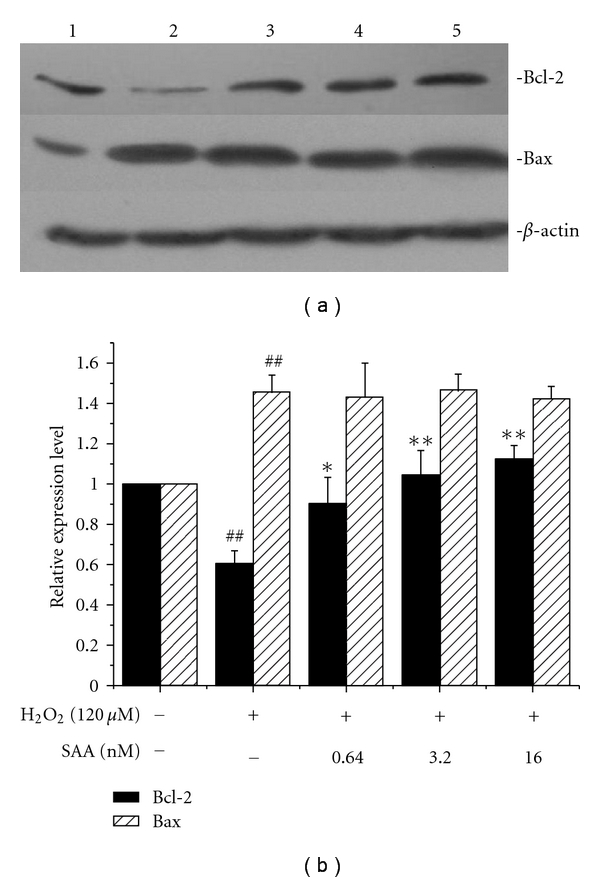
Effect of salvianolic acid A (SAA) on expression of Bcl-2 and Bax in H9c2 cells treated with H_2_O_2_. H9c2 cells were pretreated with or without SAA for 24 h prior to incubation of cells with H_2_O_2_ for 1 h. Thereafter, proteins were extracted and the protein levels of Bcl-2 and Bax were determined by Western blot analysis. Lane 1: control; lane 2: H_2_O_2_ (120 *μ*M); lane 3: H_2_O_2_ (120 *μ*M) + SAA 0.64 nM; lane 4: H_2_O_2_ (120 *μ*M) + SAA 3.2 nM; lane 5: H_2_O_2_ (120 *μ*M) + SAA 16 nM. Data are expressed as mean ± SD of three independent experiments. ^##^ *P* < .01 versus control; *P < .05, **P < .01 versus H_2_O_2_.

In the present study, H_2_O_2_-induced apoptosis accompanied decreased expression of Bcl-2 protein and increased Bax expression. Pretreatment with SAA significantly prevented the reduction of Bcl-2 expression, whereas it had no effect on Bax expression. Our results indicated that SAA exerted antiapoptotic effect through increasing Bcl-2 expression (Figure 6).

### 3.6. Effect of SAA on Phosphorylation Proteins That Are Involved in Apoptosis

 Altogether, our Phosphospecific antibody microarray detected the protein phosphorylation status of 110 sites. Using a cutoff ratio of 1.2 and 0.8 [12], we indentified 60 sites with changes in phosphorylation status due to H_2_O_2_ treatment, in which 30 sites were hypophosphorylation. Preincubation with SAA prior to H_2_O_2_ showed 18 sites with phosphorylation changes. 

Treatment with H_2_O_2_ in H9c2 cells not only resulted in a decrease in protein kinase B (Akt) and p42/p44 extracellular signal-related kinases (Erk1/2) phosphorylation but also attenuated the phosphorylation status of other direct or indirect targets, such as IKK-*α* (Thr23), HSP27 (Ser82), Bad (Ser112), and 90 kDa ribosomal S6 kinase (p90RSK; Ser380 and Thr573). Treatment with H_2_O_2_ in H9c2 cells also resulted in hypophosphorylation of other serine/threonine sites. Of these, cAMP-dependent protein kinase A (PKA; Thr197), Bcl-2 (Thr56 and Ser70), caspase-3 (Ser150), and SAPK/JNK (Thr183 and Tyr185) are known to be involved in apoptosis. Pretreatment with SAA contributed to an increase in phosphorylation (Table 1). 

Treatment with H_2_O_2_ also resulted in hyperphosphorylation of several proteins, for example, Ask1 (Ser83 and Ser966), c-Jun N-terminal protein kinase 1/2/3 (JNK 1/2/3; Thr183/Tyr185), FKHR (Ser319), and PTEN (Ser380). Ask1 and FHKR were direct Akt targets. Pretreatment with SAA decreased the phosphorylation (Table 1). 

From these results, proteins with altered phosphorylation were mainly involved in two antiapoptotic signaling pathways that were phosphatidylinositol-3-OH kinase-Akt (PI3K/Akt) and Erk1/2 pathway.

## 4. Discussion

It is widely accepted that oxidative stress, which is associated with increased formation of ROS, plays a major role in the pathogenesis of ischemia-reperfusion injury [1, 2]. ROS includes H_2_O_2_, superoxide radical, hydroxyl radical, and peroxynitrate. Among different activated oxygen species, H_2_O_2_ plays a significant role in oxidative stress injury [13]. In addition, H_2_O_2_, MDA content, and the formation of conjugated dienes in the heart were increased in ischemia-reperfusion injury, while antioxidants such as SOD and catalase protected against these changes. Furthermore, previous study showed that oxygen free radical generation rose markedly at the first minutes of reperfusion peaking at 10 min after the onset of reperfusion, and still remained of significant higher concentration 1 h after reperfusion [14]. 

*In vivo*, we attempted to simulate the time course of clinical ischemia and reperfusion events. SAA was administered 20 min before the onset of reperfusion. The obtained result showed that SAA confers significant cardioprotection. At relatively higher dose, the protection declined. The dose-response relationship could be that SAA at dose 20 mg/kg produced a maximal pharmacological response. *In vitro*, we established H_2_O_2_-induced apoptosis in H9c2 rat cardiomyoblasts to evaluate SAA's effect. Results from this study indicated that SAA effectively protected against H9c2 cell death in a dose-dependent manner within certain concentration (ranging from 0.64 to 16 nM). The dose-response relationship was consistent with that we observed in *in vivo* study. In most cases, the dose-response relationship of natural agents was not linear. It may be a bell-shaped or irregular curve. Similarly, SAA significantly increased the activity of SOD in H9c2 cells, which defended cells against H_2_O_2_ damage. Additionally, SAA prevented H9c2 cells from apoptosis at an early stage and increased antiapoptotic Bcl-2 expression by Western blot analysis. Bcl-2 is a well-known protein which potently inhibits apoptosis. All these results suggest that SAA possesses characteristics to protect myocardium from oxidative damage via antiapoptotic activity, and the protection is associated with its up-regulation of antiapoptotic Bcl-2 expression. 

To explore the antiapoptotic signaling pathway that SAA may involve, a phosphor-specific antibody microarray was used to compare the phosphorylation status, since protein phosphorylation is essential in the cellular signaling transduction and biological processes. For phosphorylation antibody arrays, each slide consists of an array of well-characterized antibody with six replicates and multiple positive and negative controls to maximize data reliability. Its efficiency and validity have been confirmed by several studies [12, 15, 16]. 

 Our Phosphospecific antibody microarray demonstrated changes in phosphorylation sites of some proteins after H_2_O_2_ treatment. The induction of phosphorylation was approximately close to control after pretreatment with SAA. It was found that SAA affected phosphorylation proteins related to PI3K/Akt and Erk1/2 signaling. 

PI3K/Akt and Erk1/2 are the antiapoptotic prosurvival kinase signaling cascades. Activation of these prosurvival kinase cascades has been shown to confer protection against reperfusion-induced injury at the time of reperfusion [17]. In Phosphospecific antibody microarray assay, changes in phosphorylation sites of some proteins related to PI3K/Akt cascade, for example, Akt, IKK-*α*, FKHR, were observed, in which IKK-*α* and FKHR have been identified as direct Akt targets [18]. As with Akt, Erk1/2 regulates apoptosis at multiple sites [19–21]. In this study, H_2_O_2_ treatment decreased phosphorylation of ERK. Pretreatment with SAA caused an increase in phosphorylation of ERK at Thr202 and Tyr204. The phosphorylated sites were consistent with the previous studies [22, 23]. 

The phosphor-antibody microarray-based studies not only gave a comprehensive insight into what had happened in the apoptotic cells induced by H_2_O_2_ but also revealed potential sites of action and signaling pathways. As many proteins are phosphorylated on multiple sites and it is the combination that determines the overall activity, further studies need to dissect causative effects from the date. Although SAA affected a number of phosphorylation proteins, we need to carry out more relative works to determine the premise mechanism of antiapoptosis of SAA. Importantly, these data provide us with information for subsequent research. 

In conclusion, these findings support that SAA possesses characteristics to decrease infarct size following myocardial ischemia-reperfusion injury and attenuate H_2_O_2_-induced apoptosis. The cardiac protection of SAA may contribute to its ability to reduce oxidative stress-induced cell damage and apoptosis.

## Figures and Tables

**Table 1:** Protein factors whose phosphorylation status altered in H_2_O_2_-induced H9c2 cells. H9c2 cells were pretreated with or without SAA (16 nM) for 24 h prior to incubation of cells with H_2_O_2_ for 1 h. Thereafter, proteins were extracted and analyzed. The phosphor-antibody microarray identified a list of protein factors whose phosphorylation status altered in H_2_O_2_-induced H9c2 cells treated with or without SAA. The signal intensities of phosphorylated proteins and the total proteins were determined. The ratio of each protein was determined as the ratio between the percentages of phosphorylated proteins of two groups. The phosphorylation induction ratio of the control group was given a value of 1.

Phosphorylation sites	Ratio (H_2_O_2_/Control)	Ratio (H_2_O_2_ + SAA/Control)	Ratio (H_2_O_2_ + SAA/H_2_O_2_)
Increase in phosphorylation due to H_2_O_2_ treatment			
ASK1 (p-Ser83)	2.09	1.10	0.53
ASK1 (p-Ser966)	1.85	1.03	0.56
B-RAF (p-Ser446)	2.23	0.79	0.35
Chk1 (p-Ser280)	2.25	0.90	0.40
Chk1 (p-Ser317)	1.59	0.94	0.59
Chk2 (p-Ser516)	2.68	1.21	0.45
c-Jun (p-Thr91)	1.34	0.88	0.66
FADD (p-Ser194)	1.26	1.16	0.92
FKHR (p-Ser319)	2.78	1.02	0.37
FOXO1/3/4-PAN (p-Thr24/Thr32)	2.45	1.06	0.43
FOXO1A (p-Ser329)	2.82	1.01	0.36
HSP27 (p-Ser15)	1.89	0.85	0.45
I*κ*B-alpha (p-Ser32/Ser36)	2.29	1.11	0.48
IKK-beta (p-Tyr199)	1.79	0.87	0.49
IKK-gamma (p-Ser85)	2.32	1.03	0.44
JNK1/2/3 (p-Thr183/Tyr185)	2.59	0.93	0.36
Lamin A/B (lamin A/C) (p-Ser392)	1.58	0.77	0.49
MKK7/MAPK7 (p-Ser271)	1.91	0.76	0.40
NF-*κ*B-p65 (p-Thr254)	1.73	0.78	0.45
NF-*κ*B-p105 (p-Ser927)	1.45	1.41	0.97
NF-*κ*B-p65 (p-Ser276)	1.43	0.79	0.56
NF-*κ*B-p65 (p-Ser536)	2.57	0.99	0.38
NF-*κ*B-p100/p52 (p-Ser865)	2.62	0.99	0.38
NF-*κ*B-p65 (p-Ser468)	2.21	0.73	0.33
PI3-kinase p85-subunit alpha/gamma (p-Tyr467/Tyr199)	2.57	1.00	0.39
PTEN (p-Ser380)	2.18	0.92	0.42
p53 (p-Ser392)	2.88	0.96	0.33
p70S6K (p-Thr229)	1.31	0.91	0.70
p70S6K-beta (p-Ser423)	1.67	0.87	0.52
TAK1 (p-Thr184)	1.60	0.98	0.61

Decrease in phosphorylation due to H_2_O_2_ treatment			
AKT (p-Ser473)	0.77	1.09	1.42
AKT1 (p-Thr72)	0.79	1.09	1.39
Bcl-2 (p-Thr56)	0.78	0.96	1.23
Bcl-2 (p-Ser70)	0.70	0.98	1.41
BAD (p-Ser112)	0.79	1.07	1.35
BID (p-Ser78)	0.61	1.27	2.10
B-RAF (p-Thr598)	0.66	1.05	1.59
Caspase-3 (p-Ser150)	0.71	0.92	1.30
Caspase-8 (p-Ser347)	0.65	1.00	1.54
CaMK II (p-Thr286)	0.66	0.93	1.41
CDK1/CDC2 (p-Thr14)	0.79	0.94	1.19
Chk1 (p-Ser286)	0.61	1.02	1.68
Chk2 (p-Thr383)	0.69	1.12	1.61
c-Jun (p-Ser243)	0.68	0.98	1.44
ERK1-p44/42 MAP Kinase (p-Thr202)	0.48	0.94	1.97
ERK1-p44/42 MAP Kinase (p-Tyr204)	0.78	0.99	1.27
HSP27 (p-Ser82)	0.79	1.02	1.28
HSP90-beta (p-Ser226)	0.65	0.81	1.23
IKK-alpha (p-Thr23)	0.75	0.95	1.26
I*κ*B-epsilon (p-Ser22)	0.74	0.92	1.26
IKK-gamma (p-Ser31)	0.33	1.04	3.18
NF-*κ*B-p65 (p-Ser311)	0.62	1.07	1.73
NF-*κ*B-p105/p50 (p-Ser907)	0.76	0.92	1.20
NF-*κ*B-p105/p50 (p-Ser932)	0.61	1.03	1.69
p70S6K (p-Ser411)	0.72	1.05	1.45
p90RSK (p-Ser380)	0.78	0.89	1.15
p90RSK (p-Thr573)	0.72	0.93	1.29
PKA CAT (p-Thr197)	0.62	1.02	1.63
SAPK/JNK (p-Thr183)	0.67	0.93	1.39
SAPK/JNK (p-Thr185)	0.64	1.02	1.58
